# War Wastage

**Published:** 1916-03-11

**Authors:** 


### "War Wastage."

WE have received a letter from the Vice-Chairman of the Prince of Wales's Hospital, objecting to the note "War Wastage," in our issue of 26th ult. His main points are that:—

"This hospital is worked under a scheme authorised by "the Board of Charity Commissioners for England and "Wales, by which the Governors are bound; and in carry-"ing out that scheme they were obliged to send out the "notices referred to (which cost the extravagant amount "of 18s. 6d.), as well as the proxy form which was of "the 'unnecessarily large size' of 8 inches by 6½ inches, "and cost 8s. 3d. Hospitals surely have the right to "expect the support and help of all papers devoted to "hospital interests, rather than such unjust criticism."

[The Prince of Wales's Hospital has had the continuous support of THE HOSPITAL for many years, and should take a little needed criticism in better part. The time is approaching, if it has not already arrived, when an independent inquiry into its administration may yield important results.—ED. THE HOSPITAL.]

